# Supplementary material for: Type XVIII Collagen Modulates Keratohyalin Granule Formation and Keratinization in Oral Mucosa
Source: Int J Mol Sci. 2019 Sep 24;20(19):4739. doi: 10.3390/ijms20194739 (PMC6801805; doi:10.3390/ijms20194739)
Supplement: Supplementary file 1 [file ijms-20-04739-s001.pdf]

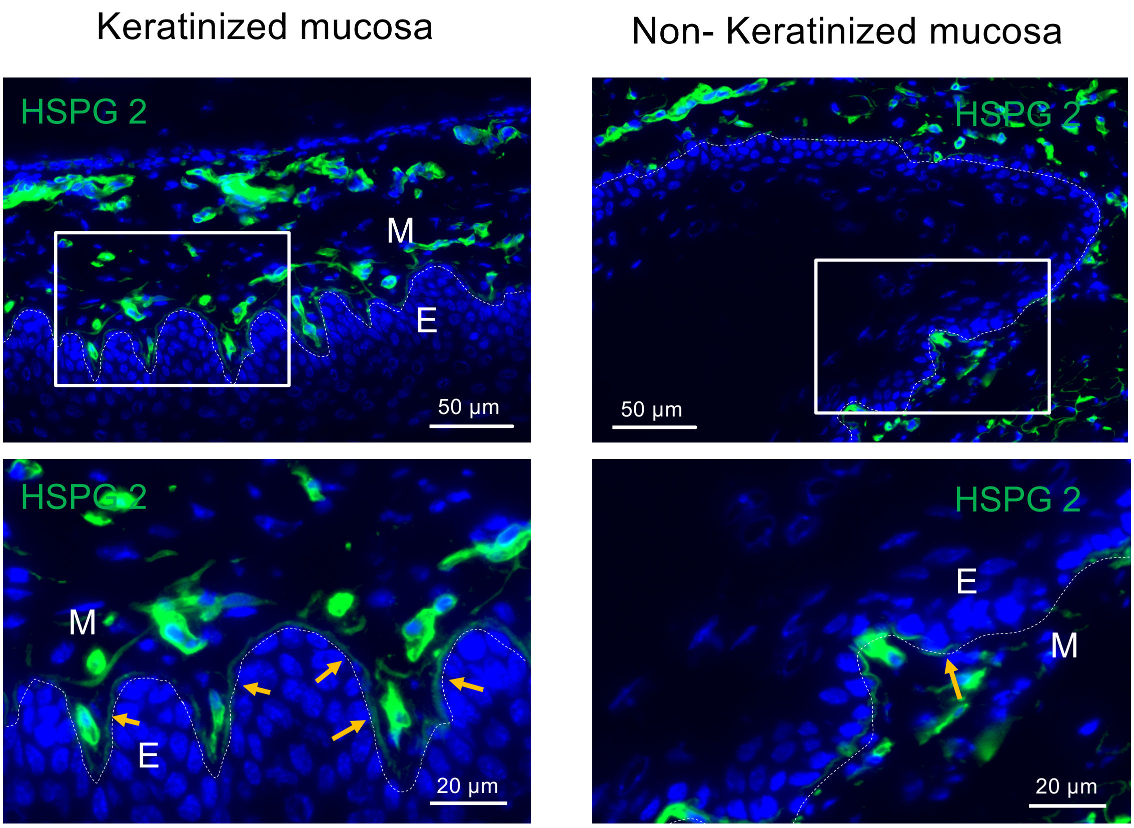

Supplemental Figure 1.
Immunohistochemical staining of perlecan in BM of keratinized mucosa and non-keratinized mucosa.
All sections were cut in the coronal direction. Boxes indicate the area shown at higher magnification in the low panels. Yellow arrows indicate the positive signal of perlecan. Note that expression level of perlecan are highly expressed in keratinized mucosa. Nuclei were counterstained with DAPI (blue). E, epithelial tissue; M, mesenchymal tissue. Results are representative of three independent experiments.

Nguyen et al.
